# Diagnostic Specificity of Two Dengue Virus IgG ELISAs after Yellow Fever and Japanese Encephalitis Virus Vaccination

**DOI:** 10.3390/tropicalmed8010007

**Published:** 2022-12-22

**Authors:** Isabelle Schnabel, Sophie Schneitler, Tom Schüttoff, Henning Trawinski, Christoph Lübbert, Christian Jassoy

**Affiliations:** 1 Institute for Medical Microbiology and Virology, University Hospital and Faculty of Medicine, University of Leipzig, 04103 Leipzig, Germany; 2 Division of Infectious Diseases and Tropical Medicine, Department of Medicine, University Hospital and Faculty of Medicine, University of Leipzig, 04103 Leipzig, Germany; 3 Institute of Medical Microbiology and Hygiene, Saarland University, Kirrberger Straße, Building 43, 66421 Homburg, Germany

**Keywords:** dengue virus, flavivirus, ELISA, antibody cross-reaction, diagnostic specificity

## Abstract

Dengue virus (DENV) antibody assays frequently cross-react with sera from individuals who have been infected with or vaccinated against related flaviviruses. The goal of this study was to determine the specificity of two DENV ELISAs with sera from individuals vaccinated against yellow fever virus (YFV) and Japanese encephalitis virus (JEV). The Panbio and the Novatec Dengue IgG ELISAs were tested with sera obtained 3–4 weeks or 0.5–6 years after YFV or JEV vaccination and the diagnostic specificity of the assays was determined. As controls, the sera were tested using DENV, YFV, JEV, Zika and West Nile virus neutralization assays. The diagnostic specificity of the Panbio and the Novatec ELISA with sera from YFV-vaccinated subjects was 98.2% and 88.2%, respectively. Cross-reactions were rare in the first 4 weeks despite high YFV-neutralizing antibody titers and were mostly found later. The specificity of the Panbio and Novatec assays with sera from JEV-vaccinated individuals was 100% and 92.9%. Cross-reactions occurred in the early time period after vaccination. The measurement values of the two ELISAs correlated strongly. Thus, the Panbio ELISA showed higher diagnostic specificity and may be suitable for seroprevalence studies in areas with high disease prevalence.

## 1. Introduction

The dengue viruses (DENVs) comprise four pathogenic flaviviruses that are transmitted by mosquitoes. The viruses cause dengue fever and severe dengue and are widely distributed in the tropics and subtropics. It was estimated that DENVs cause more than 300 million infections annually [1], but in many countries, the prevalence and incidence of the infections are unknown [2].

The prevalence of viral infections can frequently be measured by the presence of virus-specific IgG antibodies in serum samples in a group of individuals. The situation is more complicated in the case of dengue, because antibody assays for DENVs frequently cross-react with antibodies formed after infection with related flaviviruses. This makes seroprevalence studies of DENV infection challenging. Related flaviviruses that are endemic in areas of dengue fever prevalence are yellow fever virus (YFV), Zika virus (ZIKV), Japanese encephalitis virus (JEV) and West Nile virus (WNV), as well as other viruses of more regional significance. 

YFV is endemic in monkeys in tropical regions of Africa, and in Central and South America. The virus causes sporadic infections in humans and can cause disease outbreaks when the virus is transmitted between humans in urban cycles. In many endemic regions and countries, the population is routinely vaccinated in infancy or early childhood. In addition, vaccination against yellow fever is required for travelers to some of these countries [3]. JEV is endemic in parts of Central and East Asia and Oceania. The virus is transmitted to humans by Culex mosquitoes. The natural reservoir of the virus is birds. Pigs can act as amplifying hosts. Humans are dead-end hosts that are incidentally infected. Several countries in South-East Asia and Australia have public health Japanese encephalitis vaccine programs [4]. Vaccination is also recommended for travelers when a higher chance of exposure to virus-transmitting mosquitoes or risk of neurological disease is expected. 

Several studies previously described cross-reactions with commercial DENV antibody assays [5,6,7,8,9,10,11,12]. At the same time, a recent review article pointed out the limited knowledge of the frequency of cross-reactions [13]. Therefore, the aim of this study was to determine the specificity of two commercial DENV IgG antibody-binding ELISAs with sera from individuals vaccinated against YFV or JEV in comparison to virus neutralization. Since after an infection or immunization, antibody affinity matures over several months, the assays were examined with sera from 3–4 weeks or more than 6 months after vaccination.

## 2. Materials and Methods

### 2.1. Participants and Sera

Four groups of adults aged 18–60 years who were vaccinated against YFV or JEV because of travel plans to endemic areas were enrolled in this study. The participants were recruited at the outpatient clinic of the Infectious and Tropical Disease Unit at the University Hospital of Leipzig, Germany. The first YFV group consisted of 20 individuals tested 3–4 weeks after YFV vaccination (Stamaril^®^, containing live-attenuated YFV strain 17D-204, Sanofi-Pasteur). The second YFV group contained 39 individuals tested 4–6.7 years after YFV vaccination. The first JEV group consisted of 14 individuals tested 3–4 weeks after the second dose of JEV vaccination (Ixiaro^®^, inactivated JEV, strain SA_14_-14-14-2, Valneva Austria GmbH, Wien, Austria). The second JEV group contained 17 participants tested 0.5–6 years after JEV vaccination (two doses). Participants with a known episode of dengue fever, who had stayed in a dengue-endemic region for more than three months, and who had undergone previous vaccination against tick-borne encephalitis, YFV or JEV were excluded. The Ethics Committee of the University of Leipzig approved the study and the participants gave written informed consent. Blood was drawn from the participants, and sera (2–3 mL) were obtained, aliquoted and frozen at −20 °C until further use.

### 2.2. Neutralization Assays

Neutralization titers for YFV, JEV, ZIKV and WNV were determined via microneutralization assay. The sera were serially diluted from 1:20 to 1:1280 and mixed with 8–32 TCID_50_ of YFV (strain 17D-204, Genbank no. KF769015.1), JEV (Nakayma, Genbank no. EF571853.1), WNV (NY-1999, Genbank no. AF202541.1) or ZIKV (B5, Asian lineage) in 96-well cell culture plates in duplicate. Vero VFM cells (2 × 10^4^) were added and the plates were incubated for 3–4 (WNV) or 7–8 (YFV, ZIKV, JEV) days. The culture medium was removed, and cells were washed with phosphate-buffered saline (PBS) and fixed with ice-cold methanol. Afterwards, the wells were treated with PBS containing 3% bovine serum albumin as a blocking solution for 20 min. The cells were stained with the anti-flavivirus antibody 4G2 (YFV, JEV, WNV, 2 µg/mL) or ZIKV capsid antibody E4-57 (1–2 µg/mL) for one hour, washed and incubated with HRP-conjugated rabbit anti-mouse IgG antibody (Dako P0260). The plates were again washed with PBS, and TMB substrate was added to the wells. The enzymatic reaction was stopped by adding 1 N H_2_SO_4_. All incubations were conducted at room temperature. Color development was measured using a photometer at a wavelength of 450 nm. The 50% neutralizing antibody titer (NT_50_) was determined via the Spearman–Kärber equation [14]. NT_50_ values ≥ 10 and <20 were considered equivocal. NT50 values below 10 were regarded as non-neutralizing.

The sera were tested via DENV microneutralization tests in a two tier approach comprising a screening and a confirmation step [15]. For neutralization screening, the sera were diluted 1:20 and mixed with 8–32 TCID_50_ of DENV-1 isolate 2522/10, DENV-2 isolate 3229/11, DENV-3 isolate 3140/09 or DENV-4 isolate 3274/0 (provided by J. Schmidt-Chanasit, Bernhard Nocht Institute, Hamburg) in 96-well cell culture plates in triplicate. Vero VFM cells (2 × 10^4^) were added and the plates were incubated for 7–8 (DENV-2, -3, -4) or 13–14 days (DENV-1). The assay was continued as described above using the mAb 4G2. Sera that inhibited DENV infection in one or more wells were considered neutralizing or equivocal and were retested to determine the neutralization titer as described above for the other flaviviruses. Sera that did not block DENV infection in any of the three replicate wells were not neutralizing.

### 2.3. Dengue IgG ELISAs

The serum samples were tested via the Panbio Dengue IgG Indirect ELISA (Abbott Alere, Inc., Waltham, MA, USA) and the NovaLisa Dengue IgG ELISA (Novatec Immundiagnostica GmbH, Dietzenbach, Germany, Gold Standard Diagnostics Europe) according to the instructions of the manufacturer. According to the instructions for use, the Panbio ELISA uses a recombinant antigen from the four dengue virus serotypes. The NovaLisa assay uses purified DENV-2 (strain 16681). The sera were tested in duplicate. Sera from different time points after vaccination were tested on the same assay plate. To facilitate the comparison of the measuring values, the Panbio units were multiplied by 10. 

### 2.4. Statistical Analyses

Age distribution, neutralizing antibody titers and ELISA units from the different groups were compared using the Wilcoxon–Mann–Whitney U test; antibody concentrations in dependent groups were compared using the Wilcoxon signed-rank test; and correlations between the ELISA results were determined using the Spearman rank coefficient of correlation [16]. The 95% confidence intervals (CI) for the percentages of cross-neutralizing sera were calculated according to [17]. Differences between the paired proportions were compared using the McNemar test (www.medcalc.org, accessed 19 December 2022).

## 3. Results

### 3.1. Age and Sex Distribution of the Participants

The dengue IgG ELISAs were evaluated using sera from 59 YFV- and 31 JEV-vaccinated individuals subdivided in two YFV and two JEV groups. The YFV sera were obtained 3–4 weeks (mean 25 days) and 4–6 years (mean 5.36 years) after YFV vaccination. Participants in the first group were slightly younger (mean 34.8 years) than participants in the second group (44.6 years, *p* < 0.05). The JEV sera were obtained 3–4 weeks (mean 21 days) or 0.5–6 years (mean 1.76 years) after JEV vaccination (Table 1).

### 3.2. Frequency of YFV and JEV Neutralization and Neutralization Titers

**Figure 1:**
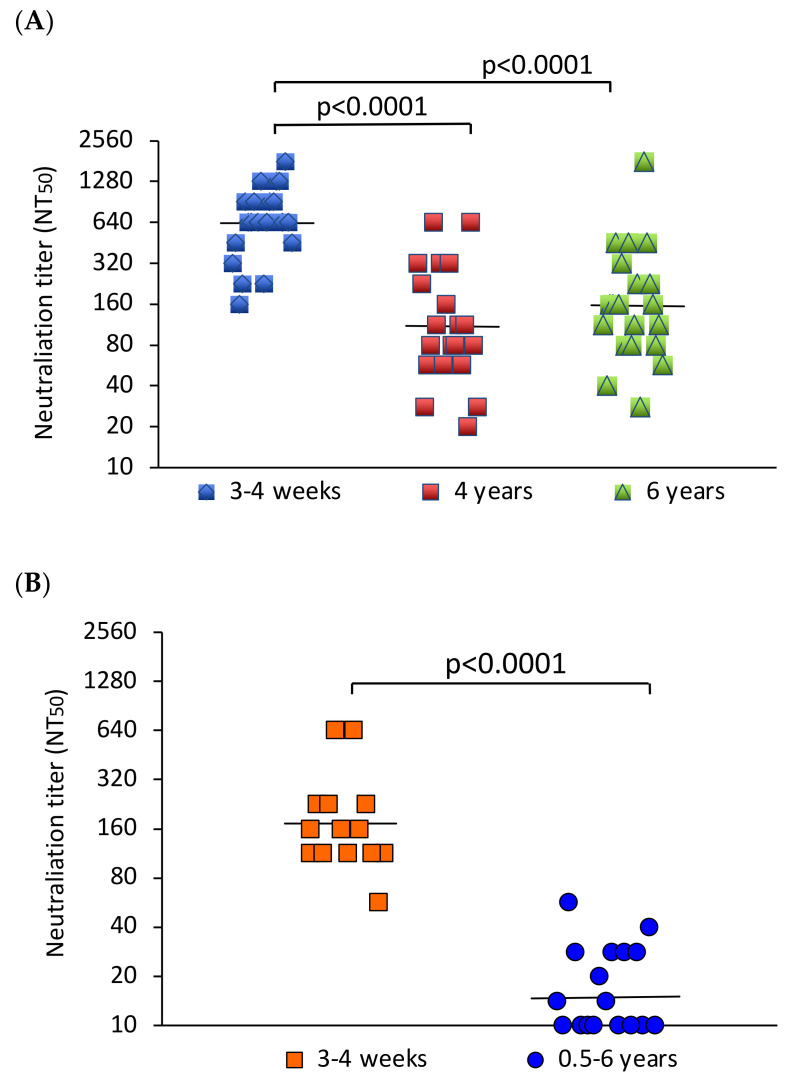
Comparison of neutralizing antibody titers early and late after vaccination. (**A**) YFV NT_50_ values of participants examined 3–4 weeks (N = 20), 4 (N = 20) or 6 years (N = 19) after vaccination. (**B**) JEV NT_50_ values of participants tested 3–4 weeks (N = 14) or 0.5–6 years (N = 17) after vaccination. The bars indicate the geometric mean neutralizing antibody titers. The values were compared using the Mann–Whitney U test. *p*-values indicate the level of significance of differences between groups.

All sera from the YFV-vaccinated individuals neutralized YFV in the microneutralization assay. The geometric mean neutralizing antibody titer (GMT) 3–4 weeks after YFV vaccination was 640. The GMTs in the groups examined 4 and 6 years after vaccination were 111 and 157, respectively. The neutralizing antibody response early after vaccination was significantly higher than that after 4–6 years (*p* < 0.0001) (Figure 1A). Ten of the freshly vaccinated individuals were followed up for 6 months after immunization. During this period, the GMT decreased slightly (Supplementary Figure S1).

In comparison, all sera from the freshly JEV-vaccinated participants neutralized JEV, and 9 out of 17 in the group tested 0.5–6 years after vaccination neutralized the virus. The GMT of the fourteen sera obtained 3–4 weeks after JEV vaccination was 172. The GMT of the sera from earlier vaccination was 15. This difference was statistically significant (*p* < 0.0001) (Figure 1B).

### 3.3. Frequency of DENV, ZIKV and WNV Cross-Neutralization

**Figure 2:**
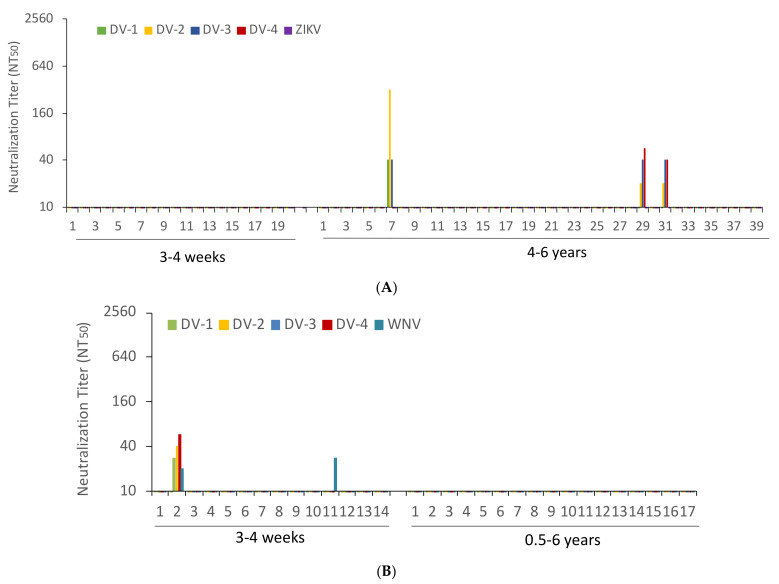
Dengue, zika and West Nile virus cross-neutralizing antibody titers. (**A**) Dengue (DV) and zika virus (ZIKV) NT_50_ values obtained with the sera of patients tested 3–4 weeks (N = 20) and 4–6 years (N = 39) after YFV immunization. (**B**) Dengue (DV) and West Nile virus (WNV) NT_50_ values of sera of patients tested 3–4 weeks (N = 14) and 0.5–6 years (N = 17) after JEV vaccination.

The sera from YFV-vaccinated participants were tested for cross-neutralization of the four DENV species and ZIKV. None of the sera from freshly YFV-vaccinated subjects neutralized any of the DENVs. In comparison, 3 of the 39 sera from 4 to 6 years after YFV vaccination neutralized DENVs. None of the sera neutralized ZIKV (Figure 2A). The sera from vaccinated individuals followed up for 6 months did not neutralize DENVs or ZIKV (data not shown). The sera from JEV-vaccinated subjects were examined for cross-neutralization against DENVs and WNV. Just 1 of the 14 sera from recently vaccinated participants neutralized DENV and WNV. A second individual neutralized WNV alone. None of the sera from individuals vaccinated more than six months before neutralized DENV or WNV (Figure 2B). Together, 3 of the 39 sera from formerly YFV-vaccinated individuals and 1 serum sample of the 14 who had undergone recent JEV vaccination contained DENV-neutralizing antibodies.

### 3.4. Diagnostic Specificity of the Panbio and NovaLisa DENV IgG ELISAs with Sera from YFV- and JEV-Vaccinated Individuals

**Figure 3:**
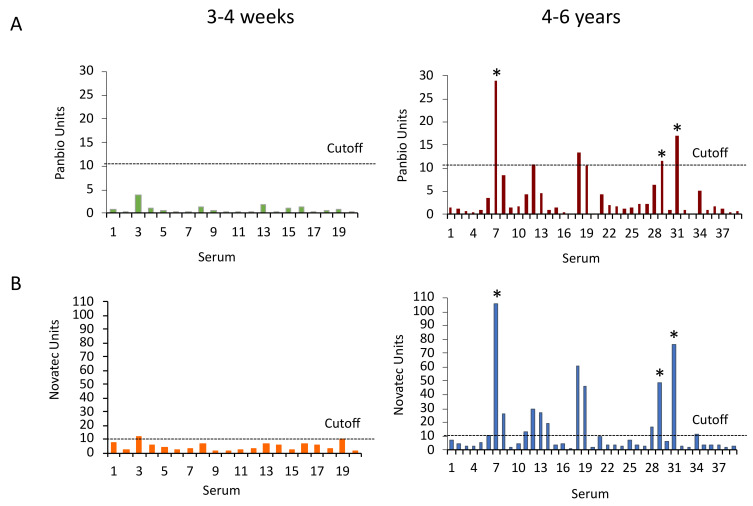
Panbio and NovaLisa Dengue IgG ELISA reactivity with sera from YFV-vaccinated individuals. (**A**) Panbio and (**B**) NovaLisa ELISA units with sera from 3–4 weeks (N = 20) and 4–6 years (N = 39) after YFV vaccination. Original Panbio measurement units were multiplied by 10. The dotted line indicates the cut-off for positive sera (11 assay units). Asterisks show sera that neutralized DENVs.

The diagnostic specificity of the the Panbio and NovaLisa DENV IgG ELISAs was evaluated in comparison to DENV neutralization. The Panbio dengue IgG ELISA was negative with the sera obtained 3–4 weeks after YFV vaccination. It was positive with four and equivocal with two sera drawn 4–6 years after vaccination, including the three sera that neutralized DENVs. In comparison, the NovaLisa ELISA was positive with one serum and equivocal with another serum from the freshly vaccinated group, and it was positive with twelve and equivocal with two sera from the group vaccinated 4–6 years earlier including the three DENV-neutralizing sera (Figure 3). Counting equivocal results as negative, the diagnostic specificity of the Panbio ELISA with the sera from YFV-vaccinated participants was 93.3 or 98.2%, depending on whether DENV-neutralizing sera were included in or omitted from the calculation. The diagnostic specificity of the NovaLisa ELISA with these sera was 78.0 or 88.2% (Table 2). The differences between the Panbio and NovaLisa assays were statistically significant according to the McNemar test (*p* < 0.0001).

**Figure 4:**
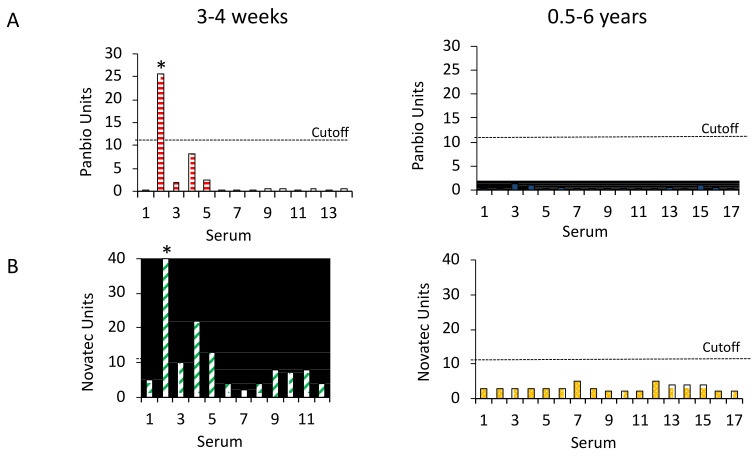
Panbio and NovaLisa Dengue IgG ELISA reactivity with sera from JEV-vaccinated individuals. (**A**) Panbio and (**B**) NovaLisa ELISA units with sera from 3–4 weeks (N = 14 and 12, respectively) and 0.5–6 years (N = 17) after JEV vaccination. The cut-off for positive sera is 11 assay units. Asterisks indicate the serum that neutralized DENVs.

The Panbio Dengue IgG ELISA reacted with 1 of the 14 sera from 3–4 weeks after the JEV vaccination. This serum also neutralized DENV viruses and WNV. The assay was negative with the 17 sera taken more than 6 months after immunization. The NovaLisa ELISA was positive with three sera including the DENV-neutralizing serum, and equivocal with one sample from the freshly JEV-vaccinated group. The assay was negative with the sera from more than 6 months after vaccination (Figure 4). The diagnostic specificity of the Panbio ELISAs with the sera from the JEV-vaccinated participants was 96.8% if the DENV-neutralizing serum was included and 100% if the serum was omitted. The diagnostic specificity of the NovaLisa ELISA was 89.7 or 92.9% (Table 2). The differences between the specificity of the ELISAs were statistically significant (McNemar test, *p* < 0.0001).

### 3.5. Correlation of the DENV ELISAs and Comparison of Values from Different Time Points after YFV Vaccination

The DENV IgG ELISA units of the Panbio and the NovaLisa assay correlated strongly with Spearman rank coefficients between 0.71 and 0.78. Thus, sera with stronger signals in the NovaLisa assay also had higher values in the Panbio ELISA, and weak signals in the NovaLisa assay were associated with low values in the Panbio assay (Supplementary Figure S2). The Panbio ELISA showed higher geometric means with sera from 4–6 years than from 3–4 weeks after YFV vaccination (*p*-value: 0.012) (Supplementary Figure S3). 

## 4. Discussion

Infection with a flavivirus frequently leads to the formation of cross-reactive antibodies against other flaviviruses. For instance, in a previous study, more than 60% of serum samples from individuals after infection with YFV or JEV cross-reacted when tested in DENV antibody immunofluorescence and ELISA assays [5]. Similarly, 11–52% of YFV-vaccinated persons cross-reacted in a DENV ELISA [6,7,8]. This makes seroepidemiological investigations about the spread of DENV infections difficult in regions with co-circulating flaviviruses and in populations that are vaccinated against YFV. The previous study results also showed that the level of cross-reaction was dependent on the IgG antibody assay and varied with the time point after vaccination. Therefore, the goal of the study was to examine and compare the diagnostic specificity of two commercial DENV IgG assays, the Panbio and the NovaLisa DENV IgG ELISA, with sera from two time periods after YFV and JEV vaccination.

We performed DENV neutralization assays to exclude previous DENV infection. None of the sera from the freshly YFV-vaccinated participants neutralized DENV, indicating that this group had not been previously infected with dengue virus. In comparison, three sera from the group of individuals who had been vaccinated 4–6 years earlier neutralized DENVs. This was most likely due to DENV infection, because DENV cross-neutralization after YFV vaccination has not been reported previously. However, since cross-neutralization could not be completely excluded, we calculated the diagnostic specificity of the ELISAs with and without DENV-neutralizing sera.

The two DENV ELISAs cross-reacted with several sera from YFV-vaccinated individuals, confirming previous reports about cross-reactions after YFV vaccination. In the Panbio ELISA, the average ELISA values were higher in the group tested 4–6 years after vaccination than in the group tested 3–4 weeks after immunization. This suggests that YFV vaccination induces the production of DENV cross-binding antibodies in a delayed way. The finding of DENV cross-reactions occurring late but not early after YFV vaccination suggests that the affinity maturation of YFV-specific antibodies causes increasing cross-reactivity with DENV. Additional studies are needed to examine how the affinity maturation of YFV-specific B-cells leads to cross-binding of the antibodies with DENV.

We also observed that the measurement values of the Panbio and NovaLisa ELISAs correlated strongly. The strong correlation of the two assays indicates that the diagnostic specificity of the two tests can easily be adjusted by raising the threshold level for positive results in the NovaLisa ELISA. Methods of optimizing the cutoff have previously been described [6,18]. In regions where people are vaccinated against YFV, an assay with a higher threshold value is advantageous.

A serum sample from the group that was freshly vaccinated against JEV neutralized the four DENVs, indicating either previous DENV infection or cross-neutralization. This participant had probably experienced DENV infection because in a previous study, DENV cross-neutralization was not observed in JEV-vaccinated individuals [19]. 

Our observations are similar to previous findings. For instance, a study by Schwartz and colleagues found DENV cross-reacting antibodies in a higher percentage of subjects more than 3 months after vaccination (44%) compared to in the first 1–2 months after immunization (15%) [8]. In the same study, the Panbio DENV IgG ELISAs cross-reacted with a fraction of sera from 1–2 months (1/10 sera) and more than 3 months (4 of 23 sera) after JEV vaccination [8]. Similarly, in a study by Bonaparte et al., the specificity of the Panbio DENV IgG ELISA was 100% in YFV-vaccinated and 97% in JEV-vaccinated individuals [20]. In comparison, in that study, 12 of 37 sera (32%) that neutralized JEV cross-reacted in a second commercial DENV IgG ELISA [20]. In another study, 12 of 16 sera from 10 JEV-infected individuals cross-reacted in a DENV IgG ELISA [21].

This study has the following limitations. The study groups were small. Therefore, a low frequency of cross-reactions with sera from individuals freshly immunized with two doses of the inactivated JEV vaccine cannot be excluded. Similarly, no data were obtained for sera from 4 weeks to 4 years and after 6 years following YFV vaccination. However, we think that the frequency of cross-reactions between 4 weeks and 4 years and after 6 years is not higher than that observed in this study. In addition, the participants were tested with vaccines available in Germany. The vaccine is widely used, but YFV vaccines based on other YFV strains are in use in some countries [22]. We would expect that the ELISAs also show cross-reactions with sera from individuals vaccinated with other YFV strains. Similarly, there are different types of vaccines against JEV including inactivated, live-attenuated and a chimeric JEV vaccine, and the recommended number of JEV vaccine doses varies [4]. The results of this study only apply to sera from individuals immunized with two doses of the inactivated JEV vaccine. A booster dose of the Ixiaro JEV vaccine is recommended if the risk of exposure persists for more than a year, and this may increase the chance of DENV cross-reactions [8,21].

In summary, the study shows that the Panbio and NovaLisa DENV ELISAs cross-reacted to varying extents with sera from individuals who received YFV and JEV vaccinations. The degree of cross-reactions depended on the vaccine, the ELISA and the time point after vaccination. The use of ELISAs for DENV seroprevalence studies in areas where people are vaccinated against YFV or JEV could lead to seroprevalence values that are erroneously too high.

## 5. Conclusions

In conclusion, a DENV IgG assay with high specificity is required in areas with regular YFV vaccination. Alternatively, the diagnostic specificity of the DENV ELISA can be increased by raising the threshold for positive results. The impact of assay specificity in DENV seroprevalence studies depends on the prevalence of the infection. For instance, in regions with low or unknown DENV prevalence but regular YFV vaccination, false positive test results may limit the usefulness of DENV ELISAs. In these situations, DENV neutralization assays are necessary for seroprevalence studies. In comparison, in areas with high DENV prevalence, an ELISA with maximum specificity is probably acceptable because the number of false positives compared to the number of true positives is small. 

## Figures and Tables

**Table 1:** Sociodemographic characteristics of the participants.

	YFV Vaccination		JEV Vaccination	
Demographic Characteristic	3–4 Weeks (n = 20)	4–6 Years (n = 39)	3–4 Weeks (n = 14)	0.5–6 Years (n = 17)
Mean age (years)	34.8 ^1^	44.6 ^1^	31.2	26.8
Range (years)	(22–50)	(18–57)	(22–51)	(21–57)
Male number (%)	8 (40)	20 (51.3)	3 (25)	5 (29.4)
Female number (%)	12 (60)	19 (48.7)	9 (75)	12 (70.6)
Time after vaccination				
Range (days)	19–28	1446–2443	21–27	187–2148
Mean	25 days	5.36 years	23 days	1.76 years

^1^ The group of freshly vaccinated individuals was statistically significantly younger (one-tailed Mann–Whitney U test, *p* = 0.00017).

**Table 2:** Diagnostic specificity of the Panbio and NovaLisa dengue IgG ELISAs.

	YFV-Vaccinated		JEV-Vaccinated	
	Panbio Dengue IgG ELISA (N = 59)	NovaLisa Dengue IgG ELISA (N = 59)	Panbio Dengue IgG ELISA (N = 31)	NovaLisa Dengue IgG ELISA (N = 29)
Positive results	4 ^1^	13 ^1^	1 ^2^	3 ^2^
Equivocal	2	3	0	1
Negative	53	43	30	25
Specificity (%, 95% CI) ^3^				
With DENV-neutralizing sera	93.3 (83.83–97.4)	78.0 (65.9–86.7)	96.8 (83.8–99.4)	89.7 (73.7–96.5)
W/o DENV-neutralizing sera	98.2 (90.6–99.7)	88.2 (70.2–90.0)	100 (88.7–100)	92.9 (77.4–98.0)

^1^ Three sera neutralized DENV; ^2^ one serum neutralized DENV; ^3^ specificity was calculated based on all sera (with) or after subtraction of DENV-neutralizing sera (w/o); equivocal results were counted as negative. CI: confidence interval.

## Data Availability

The data presented in this study are available on request from the corresponding author.

## Supplementary Materials

The following supporting information can be downloaded at: https://www.mdpi.com/article/10.3390/tropicalmed8010007/s1, Figure S1: Comparison of YFV-neutralizing antibody titers in vaccinated individuals at different time points; Figure S2: Correlation of the Panbio and NovaLisa assay results; Figure S3: Comparison of the DENV antibody units in the sera from participants vaccinated against YFV after different time periods.
