# Supplementary material for: Diagnostic Specificity of Two Dengue Virus IgG ELISAs after Yellow Fever and Japanese Encephalitis Virus Vaccination
Source: Trop Med Infect Dis. 2022 Dec 22;8(1):7. doi: 10.3390/tropicalmed8010007 (PMC9863392; doi:10.3390/tropicalmed8010007)
Supplement: Supplementary file 1 [file tropicalmed-08-00007-s001.zip › tropicalmed-2088654-supplementary.pdf]

Figure S1

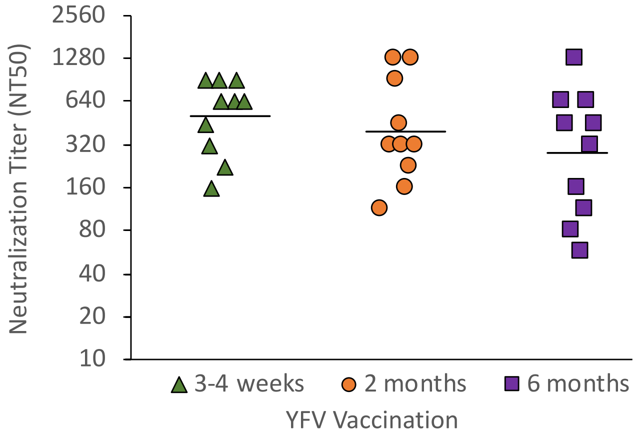

Figure S1: Comparison of YFV-neutralizing antibody titres in vaccinated individuals at different time points. YFV NT_{50} values of 10 participants vaccinated 3-4 weeks, 2 and 6 months before the measurement. The bars indicate the geometric mean titres (GMT). The values were not statistically different by the Wilcoxon signed-rank test.

Figure S2

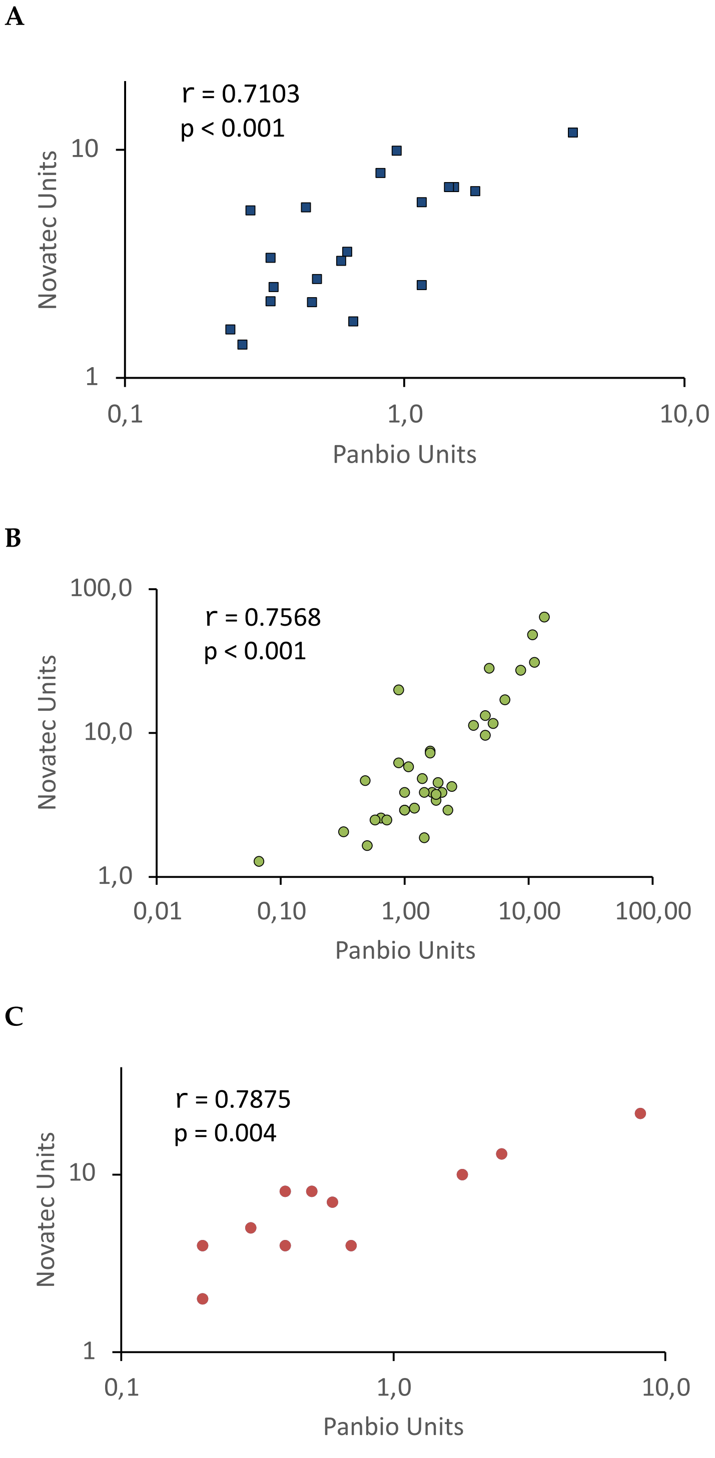

Figure S2: Correlation of the Panbio and NovaLisa assay results. **A)** Sera from individuals 3-4 weeks after YFV vaccination (N=20); **B)** Sera from individuals 4-6 years after YFV vaccination (N=36); **C)** Sera from individuals 3-4 weeks after JEV vaccination (N=11). DENV-neutralizing sera were excluded. ρ (rho): Spearman's rank coefficient of correlation; p: significance value.

Figure S3

A

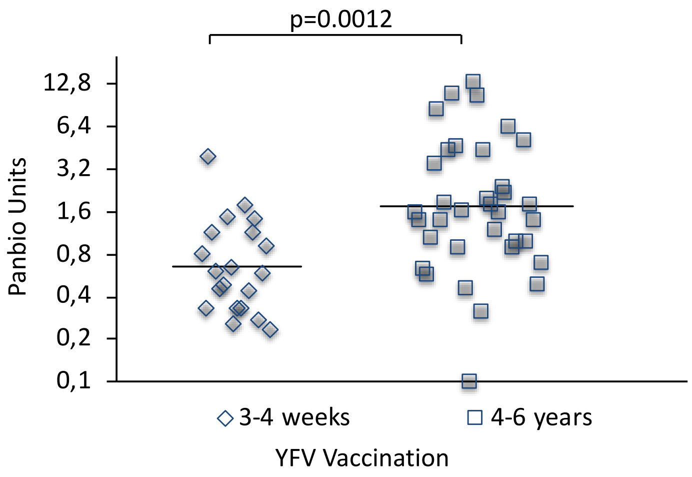

B

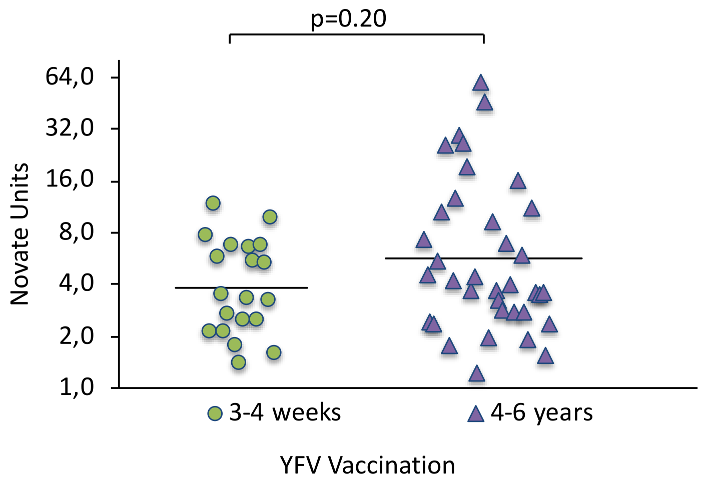

Figure S3: Comparison of the DENV antibody units in the sera from participants vaccinated against YFV after different time periods. **A)** Panbio antibody units and **B)** NovaLisa ELISA units in sera from 3-4 weeks (N=20) and 4-6 years (N=36) after vaccination. Bars indicate geometric mean values. DENV-neutralizing sera were excluded. The magnitude of the values was compared with the Mann-Whitney-U test and p indicates the level of statistical significance.
